# Myxedema Psychosis: Neuropsychiatric Manifestations and Rhabdomyolysis Unmasking Hypothyroidism

**DOI:** 10.1155/2020/7801953

**Published:** 2020-06-22

**Authors:** Sundus Sardar, Mhd-Baraa Habib, Aseel Sukik, Bashar Tanous, Sara Mohamed, Raad Tahtouh, Abdelrahman Hamad, Mouhand F. H. Mohamed

**Affiliations:** ^1^Internal Medicine Residency Program, Internal Medicine Department, Hamad Medical Corporation, Doha, Qatar; ^2^Internal Medicine Department, Hamad Medical Corporation, Doha, Qatar

## Abstract

*Background*. Hypothyroidism is a prevalent endocrine disorder, often presenting with a spectrum of symptoms reflecting a hypothyroid state. It is also generally linked to causing mood swings, psychomotor slowing, and fatigue; however, in rare instances, it may lead to or induce acute psychosis, a condition referred to as myxedema psychosis (MP). We report a case of myxedema psychosis and present a literature review discussing its presentation, diagnosis, management, and prognosis. *Case Presentation*. A 36-year-old lady presented with one-week history of persecutory and paranoid delusions, along with visual and auditory hallucinations. She had no prior history of psychiatric illnesses. She underwent total thyroidectomy three years before the current presentation due to papillary thyroid cancer. She was not on regular follow-up, nor any specific therapy. On examination, she was agitated and violent. There were no signs of myxedema, and the physical exam was unremarkable. The initial workup showed a mild elevation in serum creatinine. Additional investigations revealed a high thyroid-stimulating hormone (TSH) of 56.6 mIU/L, low free T4 < 0.5 pmol/L, elevated creatine kinase of 3601 U/L, and urine dipstick positive for blood, suggestive of myoglobinuria. MRI of the head was unremarkable. We diagnosed her as a case of myxedema psychosis and mild rhabdomyolysis. She was started on oral thyroxine 100 mcg/day, fluoxetine 20 mg daily, and as-needed haloperidol. She was closely followed and later transferred to the Psychiatry Hospital for further management. Within one week, her symptoms improved completely, and she was discharged off antipsychotics with additional scheduled follow-ups to monitor TFTs and observe for any recurrence. *Discussion and Conclusion*. Myxedema psychosis is a rare presentation of hypothyroidism—a common endocrine disorder. Scarce data are describing this entity; hence, there is currently a lack of awareness amongst clinicians regarding proper identification and management. Moreover, the atypical nature of presentations occasionally adds to a diagnostic dilemma. Thus, any patient with new-onset psychosis should be screened for hypothyroidism, and awareness of this entity must be emphasized amongst clinicians and guideline makers.

## 1. Background

When psychosis occurs as a result of a medical condition or drug, it is called secondary psychosis [1]. Amongst a variety of medical conditions, hypothyroidism can rarely lead to psychosis. This relationship was explored in 1949 by Professor Asher, and at the time, the term “myxedema madness” was coined [2]. In recent cases, the term myxedema psychosis (MP) is emerging as it better describes the condition [3, 4]. Given the rarity of the disorder, there is a significant gap in knowledge and awareness about the presentation, diagnosis, and treatment of this condition. We report the case of a young lady with myxedema psychosis and present a summary of an updated literature review with the hope of providing clinicians with a useful guide to better identify and treat this condition.

## 2. Case Presentation

We present the case of a thirty-six-year-old lady who was admitted to our hospital with a one-week history of abnormal behavior. Prior to the current presentation, she was in her usual state of health. Her employers (she works as a housemaid) stated that she had labile mood, swinging between elation (she would sing and dance), aggression, and combativeness. They also reported a history of persecutory delusions (other housemaids plotting to kill her) and hallucinations (visual and auditory).

Additionally, she developed sleep disturbance, anxious mood, and loss of appetite. She has no personal or family history of psychiatric illness. Her past medical history was significant for papillary thyroid carcinoma posttotal thyroidectomy and ablation three years before the index admission. She did not follow postsurgery and was not taking any medications. Upon her current presentation to the hospital, she was agitated and violent. Her vital signs were normal, with a blood pressure of 110/75 mmHg, temperature of 36.8°C, and a pulse of 80 beats per minute. She was oriented to time, place, and person and avoided eye contact. She looked anxious with irritable affect. Her speech was coherent and relevant, but of low tone, volume, and rate. Her answers, most of the time, were goal-directed. She had poor insight and paranoid thoughts; however, we did not elicit overt delusions. There were no findings of dry skin, voice hoarseness, nonpitting peripheral edema, or other apparent signs of hypothyroidism. Neurological examination was unremarkable, as with other systemic exams. Laboratory investigations revealed a high thyroid-stimulating hormone (TSH) of 56 mIU/mL (0.3–4.2 mIU/mL) and free thyroxine (FT4) of <0.5 pmol/L (11.6–21.9 pmol/L). Her thyroglobulin antibodies were negative. Serum creatine kinase (CK) was elevated at 3601 *μ*/L (26-192 *μ*/L), associated with a rise in serum creatinine of 111 *μ*mol/L (44-80 *μ*mol/L) and myoglobinuria. AST was 66 *μ*/L (reference range: 0-32 *μ*/L), and vitamin B12 level was normal (Table 1).

Cranial magnetic resonance imaging (MRI) was unremarkable. In summary, she had clear evidence of hypothyroidism with new-onset psychosis. After excluding plausible causes that may explain her presentation, we diagnosed the patient as a case of acute psychosis related to hypothyroidism (myxedema psychosis). Given the likely diagnosis and our previous experience with a similar case [5], we initiated oral therapy with thyroid hormone replacement (L-thyroxine 100 *μ*g/day). Our colleagues from psychiatry suggested adding fluoxetine 20 mg daily and haloperidol only as needed for agitation. Her symptoms settled within days, and she was almost at baseline within a week of therapy. The psychiatry team followed her closely, and later, we transferred her under their care in the Psychiatry Hospital for further observation. She remained there for one week and was discharged home off antipsychotics. Upon discharge, she demonstrated normal mood, insight, and mental status; she was advised to follow the thyroid function test and adhere to thyroxine replacement. The patient returned to her home country, and unfortunately, she was lost to follow-up thereafter; thus, information regarding medication compliance, adverse effects, or any further relapses of myxedema psychosis is not available.

## 3. Discussion

Our patient was diagnosed with an uncommon manifestation of hypothyroidism referred to as myxedema madness or better termed myxedema psychosis. It is a form of secondary or organic psychoses. This condition was described in 1949 by Professor Asher in a study of fourteen patients with psychosis and hypothyroidism. It was him who named the condition myxedema madness and provided us with the first demographic characterization of this condition [2]. However, the association between psychosis and hypothyroidism has long been described for more than a century [6]. A survey conducted by the Committee on Myxedema of the Clinical Society of London in 1888 confirmed one hundred and nine patients with myxedema and reported that more than half of these patients experienced hallucinations [6].

Compared with the prevalence of hypothyroidism, myxedema psychosis is thought to be rare, with only a few cases reported in the literature along with scarcity of observational or systematic studies. The inadequacy of relevant data regarding this condition perhaps led to the lack of validated standardized diagnostic tools. The exact mechanism of myxedema psychosis remains unclear; postulated theories are mainly derived from data based on animal studies. The localization of thyroid hormone receptors in the limbic structure, which is a crucial area for emotional and behavioral integration, is thought to play a role in the setting of thyroid hormone imbalance [7]. Additionally, the role of the anterior locus coeruleus tyrosine hydroxylase imbalance resulting from dysthyroid status was also described [8]. Studies from humans have demonstrated that glucose metabolism and cerebral perfusion may be reduced in this cohort of patients [9, 10].

Our knowledge about this condition is derived from sporadic case studies. It appears that the extent of thyroid dysfunction does not correlate with the degree of accompanying psychiatric manifestations [11]. Our exhaustive literature review revealed that delusions seem to prevail in this population, with the majority being persecutory, paranoid, and religious delusions. Auditory hallucinations are also common in this cohort of patients [5, 12–18]. History of hypothyroidism is supportive of MP [5]; however, it was not present in many cases [3, 5, 19]. Additionally, typical symptoms and signs of hypothyroidism are indeed helpful when present; but their absence in cases of MP has also been reported [20–23]. Furthermore, taking history in the setting of acute psychosis is challenging and often unfruitful, especially for clinicians not well-versed in this domain. Therefore, the absence of typical symptoms or signs of hypothyroidism should not preclude the diagnosis [5].

Laboratory testing helps to confirm hypothyroidism. The cerebrospinal fluid (CSF) exam is usually not performed; however, when attempted, it is generally unremarkable, while mild CSF protein elevation is also rarely reported [5, 12, 17, 23, 24]. MRI and electroencephalogram are mostly insignificant [5, 14, 23, 25, 26]. Notably, the patient had evidence of rhabdomyolysis—another infrequent association with hypothyroidism. We have previously reported a similar case presenting with the rare combination of MP and rhabdomyolysis [5]. This second case calls perhaps for future research examining this relationship by first exploring the exact prevalence of rhabdomyolysis in cases of MP. The treatment includes correcting the thyroid imbalance. It was thought to be enhanced via administering intravenous thyroxine or even triiodothyronine [3, 14, 27, 28]. However, many recent cases have shown excellent outcomes utilizing oral thyroxine (T4) [16, 21]. In most cases, psychosis responded to short-term antipsychotics and was discontinued upon follow-up [23, 29]. Interestingly, very few cases were managed using only thyroxine without the use of antipsychotics [20, 29]. The majority of patients achieved full recovery within a few days to a few weeks. Few cases were left with some deficits; this was thought to be due to untreated chronic hypothyroidism with resultant brain damage [2].

This case is compelling because it is the second presentation of the rare myxedema psychosis we encountered in our hospital. We think that our cumulative knowledge, from the previous encounter, helped us in promptly identifying and treating this case. This highlights the importance of raising awareness and providing guidance about this infrequent entity amongst frontline clinicians. Additionally, we found rhabdomyolysis concurrently with myxedema psychosis in this patient as with our first case, as explained earlier; this extremely rare association may need further exploration [5]. Our case also highlights the need for standardized criteria that can adequately help to diagnose MP and also differentiate it from other mimickers, such as Hashimoto's encephalopathy.

## 4. Conclusion

Hypothyroidism may manifest as acute psychosis; however, diagnosis may be missed specifically in cases without a previous history of thyroid disease. In patients with acute psychosis, screening for thyroid disorders is imperative, given the wide range of presentation and the absence of validated diagnostic tools. Additionally, the fact that hypothyroidism is reversible with early treatment initiation shows promising outcomes. The management of hypothyroidism-induced psychosis includes thyroid hormone replacement and short-term antipsychotics initially until psychotic symptoms improve.

## Figures and Tables

**Table 1:** Laboratory investigations upon admission and on the fourth day of hospitalization.

Lab value	Admission	Day 4^∗^
Hemoglobin (13-17 gm/dL)	11.4	10.8
Creatinine (44-80 *μ*mol/L)	111	93
Sodium (135-145 mmol/L)	142	140
Potassium (3.5-5.1 mmol/L)	3.2	3.7
TSH (0.30-4.2 mIU/L)	56.6	ND
Thyroglobulin antibodies (<22 IU/mL)	ND	<0.9
Creatinine kinase (22-192 U/L)	3601	3129
ALT (0-33 U/L)	ND	26
AST (0-32 U/L)	ND	66

^∗^No labs were repeated afterwards. ND = not done; TSH = thyroid-stimulating hormone; ALT = alanine transaminase; AST = aspartate aminotransferase.
